# Indirect impact of violent events on emergency department utilization and disease patterns

**DOI:** 10.1186/s12873-020-0307-5

**Published:** 2020-02-13

**Authors:** Carla Makhlouf-Obermeyer, Eman Sharara, Ghada El-Eid, Eveline A. Hitti

**Affiliations:** 1 Center for Research on Population and Health, Faculty of Health sciences, American University of Beirut, Beirut, Lebanon; 2 Institute of Advanced Studies, Aix-Marseille University, Marseille, France; 3 Director Work Process Unit, American University of Beirut Medical Center, Beirut, Lebanon; 4 Department of Emergency Medicine, American University of Beirut Medical Center, Beirut, Lebanon

**Keywords:** Violent events, Health outcomes, Stress, Emergency department

## Abstract

**Background:**

The health effects of war and armed conflict on casualties and mental health of those directly exposed has been well described, but few studies have explored the indirect health effects of violent events. This paper assesses the indirect health impact of several violent events that took place in Beirut in 2013–2014 on ED visit utilization and disease patterns.

**Methods:**

As tracked by media reports, there were 9 violent events in Beirut during 2013–2014. We compared visits to the Emergency Department of a major medical center during weeks when violent events happened and weeks without such events (the preceding week and the same week in preceding years). After re-coding de-identified data from the medical records of 23,067 patients, we assessed differences in the volume of visits, severity index, and discharge diagnoses. Individual control charts were used to analyze ED visit trends post-event.

**Results:**

Comparisons of weeks with violent events and weeks without such events indicate that the socio-demographic characteristics of patients who visited the Emergency Department were similar. Patients seen during violent weeks were significantly more likely to be admitted to the hospital, and less likely to present with low acuity complaints, indicating greater complexity of their conditions. The discharge diagnoses that were significantly higher during violent event weeks included anxiety disorders, sprains, and gastritis. Daily ED visits dropped post events by 14.111%, *p* < 0.0001.

**Conclusions:**

The results indicate that violent events such as bombs, explosions, and terrorist attacks reverberate through the population, impact patterns of ED utilization immediately post-event and are associated with adverse health outcomes, even among those who are not directly affected by the events.

## Background

There is a vast literature on the health effects of war and armed conflict, and numerous reports attempt to quantify the number of deaths and casualties that they cause among general populations and armed personnel [1–6] as well as the impact on mental health of direct exposure, particularly Post-Traumatic Stress Disorder [7–10]. The other mechanisms by which violence affects health are recognized but under-studied [6], and no studies have investigated the effect of multiple discrete violent events during periods of relative calm. Investigating violent events that occur during otherwise peaceful and relatively normal times, as we do here, makes it possible to focus on the impact of the events rather than on the general disruption that accompanies armed conflicts. The availability of good medical records from a major hospital also represents an additional advantage, particularly in view of the difficulty of conducting surveys on the impact of violence under the very difficult conditions that prevail in times of conflict.

Conceptually, studies of the indirect impact of violence on health build on a long tradition of research that has documented associations between stressful life events and a variety of health outcomes, including mental health problems, cardiovascular diseases, high blood pressure, some autoimmune diseases, as well as biological markers of inflammatory disorders [11–19]. From a methodological point of view, despite the development of many instruments since the classic study of Holmes and Rahe [20], a major difficulty has been that usually, both the identification of stressful events and the assessment of their impact rely on subjects’ reports [21]. In this study, we identify nine violent events on the basis of media report happening in the most recent years (2013 and 2014) and use medical records to assess health conditions, thus measuring exposures and outcomes in an objective manner.

The literature on violent events has highlighted the challenge that multiple casualties represent for medical facilities, particularly in situations where these are insufficiently staffed due to the loss of health professionals [22], but there have been no analyses of the impact of violent events on patterns of Emergency Department (ED) visits. Surveys on individuals indirectly exposed to violent events, show a significant percentage reduction in local travel post-event because of perceived safety issues [23]. How this impacts people’s behaviors with respect to seeking care for acute medical issues post-event has not been explored. Literature on simulating flow to EDs and impact of large scale events on ED operations has been limited to planned city events [22]. Thus, the implications of this study are relevant to research on ED operations during violent events from a staffing and resource utilization standpoint.

This paper aims to assess the indirect health impact of violent events on ED visit utilization and disease patterns. Our exposure variable is the occurrence of violent events such as bombs, explosions, or armed clashes that caused destruction and/or casualties in Greater Beirut during 2013–2014. While this analysis includes casualties that were caused by explosions or armed conflicts, we are especially interested in the indirect impact of violent events on populations not directly affected by them. Our hypothesis is that when these events occurred, individuals living in Beirut were all exposed to a degree of stress, because in addition to casualties and destruction, these events cause fear about security for those living in the city, worries that the danger can escalate, and a sense of uncertainty about the future and thus might impact the types of conditions presenting to the ED as well as general ED utilization. We thus investigate visits to the Emergency Department to assess the extent to which the volume of visits, the severity of cases, and the frequencies of various discharge diagnoses differed significantly between periods when violent events occurred, compared to quieter periods.

## Methods

### Study setting

This study was conducted at the Emergency Department (ED) of a major urban teaching hospital and referral center in Beirut, Lebanon. The hospital is one of many in the Beirut area located in the center of the city, at a distance from the sites of violent events ranging between 1.4 km to 12.5 km. The ED was the most proximal site for only one of the events (Dec 27, 2013), with more proximal hospitals receiving the majority of the direct casualties for the remaining events. The ED is organized into a high acuity unit, a low acuity unit and a pediatrics unit; core ED personnel includes American Board-certified/eligible physicians in Emergency Medicine, as well as physicians without specific emergency training, but with extensive experience in emergency medicine. The number of visits to the ED is about 55,000 per year. This study was deemed exempt from human subject research by our Institutional Review Board.

### Study design

This study was designed in accordance with STROBE guidelines [see Additional file 2]. We used a variant of case-control design to compare patient acuity and disposition in weeks where events happened, compared to weeks where no events took place. For each event, we defined an “event,” or “case” week as the week starting on the day of the event. We defined two “no-event”, or “control” weeks: the week before the event and the same week in the preceding year. We assumed that events occur randomly, and that event (case) and no-event (control) weeks are similar in all respects, except the occurrence of the events.

To study the impact of events on utilization of EDs, we compared ED daily visit volumes 30 days pre-event with ED daily visit volumes 30 days post-event. For this analysis, we considered only events preceded by at least 2 months of calm to allow for patterns of ED utilization to stabilize back to non-conflict routine. Three events fit these criteria (event 1:July 9, 2013; Event 2: Nov 19, 2013; Event 3: June 24,2014) and were used for the trend analysis.

### Study protocol

In this analysis, we focused on the most recent years of violent events happening in Beirut (2013 and 2014). Additional file 1: Table S1 provided a summary of the events and the weeks included in the study [see Additional file 1].

We retrieved de-identified data from medical records for patients who visited the Emergency Department (ED) during the selected periods in 2012, 2013, and 2014. There were 7874 visits to the ED during event weeks, and 15,193 during no-event weeks, for a total of 23,067 visits for which individual medical records were reviewed. The following data were extracted from each record: age, gender, nationality, residence, admitting diagnosis, how bill was paid (a variable associated with employment and socio-economic status), length of stay, discharge information (admitted or discharged), and discharge diagnosis.

Trained staff recoded the physicians’ diagnoses to International Classification of Diseases, Ninth Revision, Clinical Modification (ICD-CM9) codes (21). To reduce the number of codes and make comparative analyses more manageable, these diagnoses were further classified into single-level categories, using Clinical Classifications Software (CCS), which is the standard used for many analyses [24]. Thus, the 1864 ICD-9-CM diagnoses were collapsed into 218 codes, and all the diagnoses used here refer to the single-level CCS categories that the ICD-9 coded diagnoses mapped to, rather than to the clinical definitions themselves.

For each patient who visited the ED during the study weeks, we also included information on the severity of the case. We used the emergency severity index (ESI), a well-validated triaging score which relies on trained nurses to make an acuity judgment based on the likelihood of immediate threats to life or organs, and to predict the number of resources that would be required in order to stream patients to appropriate care [25, 26]. A score of 1 or 2 indicates high acuity, a score of 3 intermediate, and a score of 4 or 5 low acuity; thus the scores were re-categorized into these three levels.

### Statistical analyses

Descriptive statistics were used to compare the volume of visits and patients’ characteristics for event and no-event weeks, using chi square, Fisher’s exact tests, t-tests, or Cochrane-Armitage as appropriate. We used all the variables available in the medical record to compare patients in event and no-event weeks. Logistic regression was used to assess the significance of differences in the frequencies of CCS codes between event and no-event weeks.

Individual control charts (ICR) were used to assess impact of events on ED visit trends and assess for special cause variation (non-routine events). Baseline values were computed using daily ED visit data 30 days prior to each event with control limits set at 3 standard deviations (SD) above and below the center line, using Quantum XL. Time related variation was based on 2 rules: rule 1, where 6 or more consecutive points steadily increase or decrease; rule 2, where 15 consecutive points fall within +/− 1 SD on either side of the center line. Baseline data was compared to daily ED visit data up to 30 days post-event. When a period of calm was followed by a series of back to back events that were separated by less than 1 month, the post event period included all ED visits up to 30 days post last event in the series.

In addition, daily ED visit trends were further analyzed using interrupted time-series analysis for the period 30 days before the event, and 30 days after the event; segmented regression analysis was conducted using the newey command (considering Newey-West standard errors) in STATA version 15 (StataCorp LLC., College Station,TX). Statistical significance was defined as *P* < 0.05.

## Results

Patients of all ages visited the ED during the weeks selected for this study, with the largest group being young adults. There were slightly more males (51%) than females among patients. The majority were discharged, and around 15% were admitted to the hospital (Table 1). There were no statistically significant differences between patients admitted to the ED during event and no-event weeks by gender, marital status, and nationality; some statistical differences by residence were found but they were very small. Significantly more patients who visited the ED during no-event weeks were discharged (83% compared to 81% during event weeks, *p* < 0.001). Only 36 patients were admitted to the ED as casualties from the violent events. In addition, a smaller percentage of patients who visited during event weeks had low acuity triage status than on no-event weeks (19.8 vs 27.36), while more patients had intermediate triaging status (76.64 vs 68.03, *p* < 0.001), reflecting a higher complexity of cases during event weeks (Table 1).

**Table 1:** Characteristics of patients who visited the ED during event and no-event weeks (percent)

	Total patients (%) *N* = 23,067	Event weeks (%) *N* = 7874	No-event weeks (%) *N* = 15, 193	Chi square, *p*-value
Gender				
Males	50.9	50.1	51.4	X^2^(1) = 3.14, *p* = 0.076
Age (years)				
Mean (SD)	33.96 ± 24.4	33.22 ± 24.6	33.82 ± 24.3	*t* = −1.1832, *p* = 0.2367
Disposition				
Admitted	15.2	15.8	14.9	X^2^(3) = 19.38, *p* < 0.001
Discharged home	82.13	81.0	82.7	
Dead	0.18	0.15	0.2	
Others^a^	2.49	3.05	2.2	
Severity Index (ESI)				
High Acuity	4.18	3.49	4.61	Z = -9.69, *p* < 0.001
Intermediate Acuity	71.30	76.64	68.03	
Low Acuity	24.52	19.88	27.36	

^a^Includes discharged against medical advice, incomplete service, transfer to another hospital or private clinic, and not specified

**Fig. 1:**
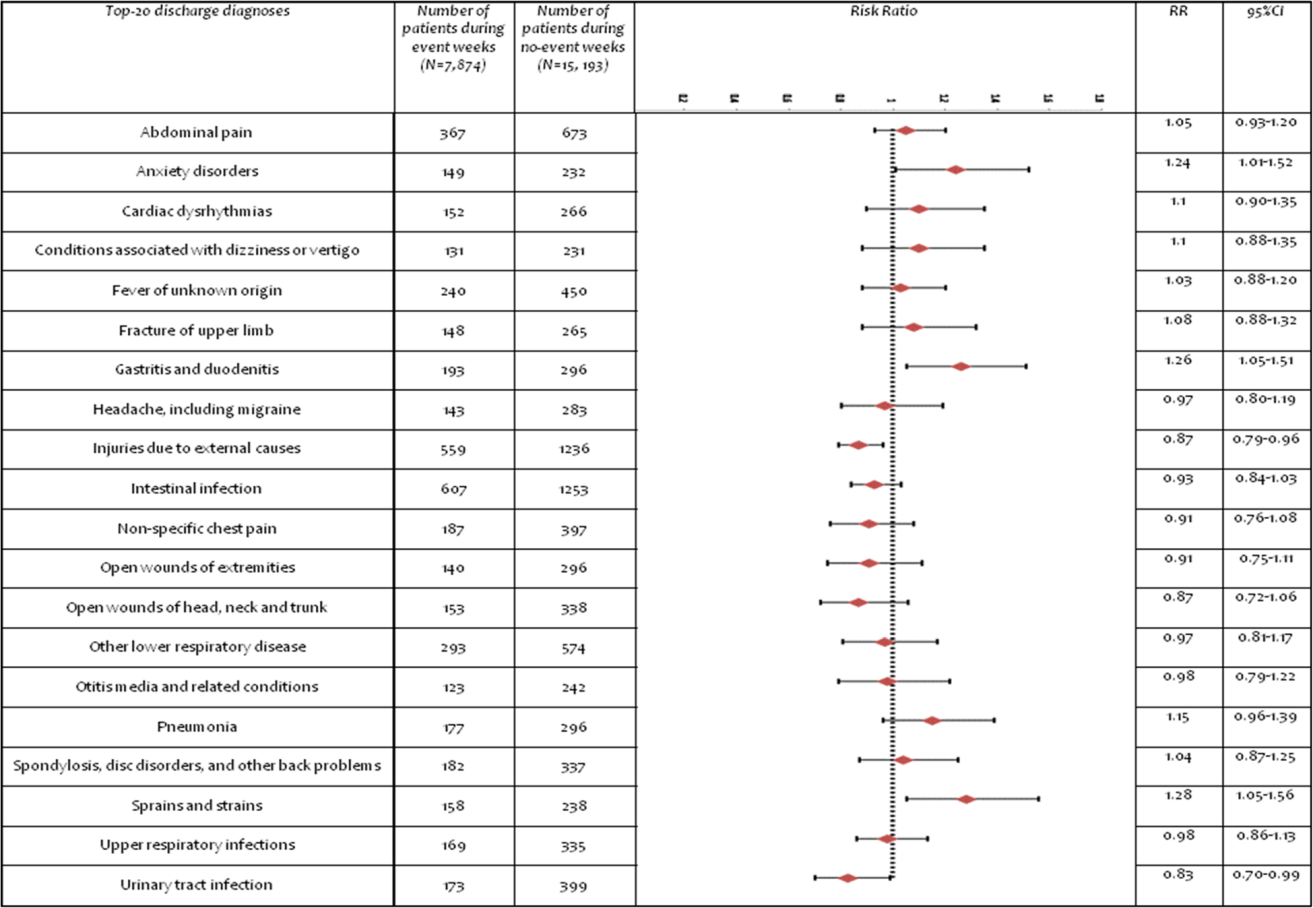
Comparison of the top-20 discharge diagnoses for event and no-event weeks: frequencies, risk ratios and confidence intervals

All discharge diagnoses were examined to assess whether there were significant statistical differences between event and no-event weeks. The frequencies of discharge diagnoses varied considerably, with some diagnoses accounting for only one case and others as many as 230; there were 140 diagnoses that accounted for 50 or fewer cases out of the total 23,067 visits (0.2%). These less frequent diagnoses accounted for a total of 9986 visits during the study period, and while a few (such as mycoses, influenza, bronchitis, eye infections, and cardiac anomalies) differed significantly between event and no-event weeks, the differences were very small, and these results are not shown. Instead, we focused on the top 20 discharge diagnoses. Figure 1 shows a comparison between event and no-event weeks, using risk ratios and confidence intervals. Patients who visited during event weeks were more likely to receive a discharge diagnosis of strains/sprains (RR = 1.28, CI = 1.05–1.56), gastritis (RR = 1.26, CI = 1.05–1.51), and anxiety disorders (RR = 1.24, CI = 1.01–1.52). They were less likely to receive a discharge diagnosis of urinary tract infection (RR = 0.83, CI = 0.70–0.99); unexpectedly, they were less likely to receive a discharge diagnosis of injuries (RR = 0.87, CI = 0.79, 0.96), a point to which we return later.

**Fig. 2:**
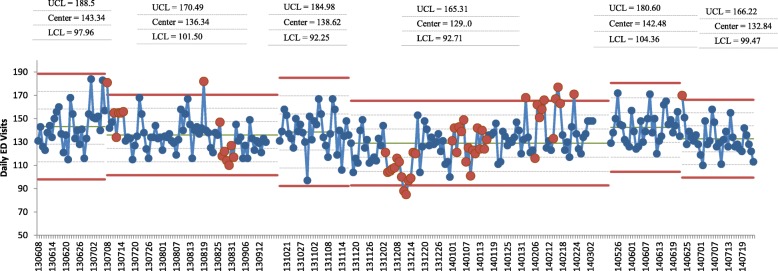
Daily ED Visit Individual Control Chart

Individual control charts showed a drop in center line in all three events that were preceded by at least 2 months of calm from 143.34.5 to 136.34 for the first event, 138.62 to 129 for the second event and 142.48 to 132.84 in the third event (Fig. 2). Special cause variations appeared in all three post-event periods, with the highest number in the second period that included multiple back to back events.

Interrupted time-series analysis showed a signifcant decrease in daily ED visits by 14.11% per day and a *p*-value of < 0.001 (Table 2).

**Table 2:** Interrupted time-series analysis of daliy ED visits pre and post event

	Change in trend	95% CI	*P*-value
Event 1	−8.07	−17.92; 1.79	0.11
Event 2	−22.53	−32.83; −12.24	< 0.0001
Event 3	−11.73	− 18.41; − 5.06	0.001
Average events	− 14.11	− 20.89; − 7.33	< 0.0001

## Discussion

Our results indicate that patients who visit the ED during weeks when violent events occur have more serious conditions and are more frequently admitted to the hospital than those who visit the ED during weeks when no such events happen. Overall ED utilization however appears to drop following events that are preceded by periods of calm.

The fact that the volume of ED visits during weeks post event is lower than pre-event weeks deserves some attention*.* Concerns about the security situation immediately post-violent event may impact patient decision to seek medical care for certain conditions and lead to delays in care except for conditions perceived as urgent by patients. This may also explain the fact that external injuries were not higher during event weeks, as individuals may have delayed care for injuries that did not seem very urgent. The relatively low number of casualties and the fact that diagnoses related to injuries (fractures, open wounds, other injuries) did not differ significantly between event and no-event weeks also have a practical explanation: most of the violent events included in this study took place in a part of the city that includes several hospitals and that is quite distant from the Medical Center where our study took place; in the absence of an Emergency Medical Services central command in Lebanon, most casualties and injuries would have been taken to nearby hospitals rather than to the Medical Center that we studied. These points underscore the importance of considering the distal effects of violence along with its direct impact; it is all the more remarkable that these effects could be detected at health facilities that were distant from the violent events themselves.

The discharge diagnoses that were found to be significantly higher during event weeks include anxiety disorders, consistent with the notion of violence-related mental stress. Our finding that gastritis and duodentis were more frequent during event weeks is consistent with the results of studies on the associations of stress with immunity and biomarkers of inflammation [11, 27]. The discharge diagnoses that were more frequently found during control weeks appeared to be less consistently associated with stress (urinary tract infection and injuries due to external causes). Taken together, these findings provide support for our hypothesis that violent events represent stressors that increase vulnerability to health conditions. Our study is consistent with other research conducted among Lebanese [28, 29], Palestinians [30], and Iraqis [6], that found that war, conflict and political unrest are associated with adverse health outcomes. Because we assessed health outcomes through the records of a well-functioning medical center during relatively peaceful times, our study suggests that it is the violent events themselves, rather than the disruption of health care, that account for the adverse health outcomes that we found.

Our results have implications for the management of emergency care after violent events. Although much has been written on emergency and hospital preparedness for receiving mass casualties during violent events [31, 32], less is known about resource implications of the indirect effect of violent events on hospitals and EDs. Understanding the impact of stressful events on population health, the types of ED diagnoses presenting to EDs and the acuity that can be expected after violent events can help hospitals and EDs better meet their community’s needs.

There are a number of limitations to our study. The time frame was limited to the week following the events, and could not capture the possible longer-term consequences of exposure to violence. Also, although our overall sample size was very large, cell sizes for particular conditions were small and this may have resulted in under-estimates of statistically significant associations. Another limitation of the study is that, as previously mentioned, the study facility was not the closest hospital to where the events occurred and therefore may not be representative of the population most impacted by the event. There are also limitations related to the management of discharge diagnosis data, since, as has been documented in other studies [33, 34] recoding physicians’ notes is a complex process requiring a great deal of training to select the appropriate code and ascertain principal vs. incidental conditions. In addition, there are trade-offs in a study design such as ours, because in the absence of reports by study participants about their experience, stress is assumed to have affected all individuals living in the city at the time. That such an assumption is justifiable is supported by observations of life in Beirut through various times of violence, and of similar violent events occurring in other countries such as 9/11 in the US or the November 2016 terrorist attack in Paris. Still, complementing “objective” measures such as we used, with participants’ reports of their experience would have enriched the project.

## Conclusions

The results indicate that violent events such as bombs, explosions, and terrorist attacks reverberate through the population, impact patterns of ED utilization immediately post-event and are associated with adverse health outcomes, even among those who are not directly affected by the events.

## Supplementary information

**Additional file 1:Table S1.** Violent events in Beirut 2013–2014, and corresponding “case” and “control weeks”.

**Additional file 2.** Assessment of the publication based on STROBE guidelines checklist.

## Data Availability

All data generated or analyzed during this study are included in this published article [Supplementary file 3].

## Abbreviations

CCS: Clinical Classifications Software
ED: Emergency Department
ESI: Emergency Severity Index
ICD-CM9: International Classification of Diseases, Ninth Revision, Clinical Modification
PTSD: Post-traumatic stress disorder
