# Supplementary material for: Indirect impact of violent events on emergency department utilization and disease patterns
Source: BMC Emerg Med. 2020 Feb 13;20:10. doi: 10.1186/s12873-020-0307-5 (PMC7020587; doi:10.1186/s12873-020-0307-5)
Supplement: Supplementary file 1 — Additional file 1:Table S1. Violent events in Beirut 2013–2014, and corresponding “case” and “control weeks”. [file 12873_2020_307_MOESM1_ESM.docx]

| **Supplementary Table 1.**  **Violent events in Beirut 2013-2014, and corresponding “case” and “control weeks”** | | | | | | |
| --- | --- | --- | --- | --- | --- | --- |
| **Date of event** | **Event description in electronic media** | **Corresponding event weeks**  (Subtotal: 62 days) | **Corresponding**  **no-event weeks**  (Subtotal: 117 days) | **Distance from event to hospital, in Km (Time from event to hospital, in minutes)** |  |  |
| **July 9, 2013** | A car bomb wounded at least 53 people in Dahiyeh, a mainly Shia neighborhood in southern Beirut. The blast occurred in the car park of a supermarket in the residential area of Bir el-Abed, causing heavy damage but no fatalities | **July 09, 2013- July 15, 2013** | **July 09, 2012-July 15, 2012**  **July 2, 2013-July 8, 2013** | **9.1 (16)** |  |  |
| **August 15, 2013** | A car-bomb explosion killed at least 20 people in a southern suburb of Beirut. At least 200 people were injured in the blast, which occurred between the Bir el-Abed and Roueiss neighbourhoods. | August 15, 2013- August 21, 2013 | August 15, 2012- August 21, 2012  August 08, 2013- August 14, 2013 | **9.1 (16)** |  |  |
| **November 19, 2013** | The Iranian embassy in Beirut is hit by a double suicide attack car-bomb, killing at least 23 people and wounding 150 others. | November 19, 2013- November 25, 2013 | November 19, 2012- November 25, 2012  November 12, 2013- November 18, 2013 | 5.4 (12) |  |  |
| **December 3, 2013** | A senior Hezbollah commander named Hassan Lakkis was assassinated by 2 gunmen in Beirut | December 3, 2013- December 9, 2013 | December 3, 2012- December 9, 2012  November 26, 2013- December 2, 2013 | 12.5 (21) |  |  |
| **December 27, 2013** | Mohamad Chatah, Lebanon's former finance minister is killed by a car bomb that hit in the centre of the capital Beirut, along with at least five others. | December 27, 2013- January 1, 2014* | December 27, 2012- January 1, 2013*  December 20, 2013- December 26 , 2013 | 1.4 (5) |  |  |
| **January 2, 2014** | A car bomb was detonated in the Haret Hreik area of the Southern suburb of Beirut, [killing](http://www.dailystar.com.lb/News/Lebanon-News/2014/Jan-06/243254-al-manar-reporter-wounded-in-beirut-bombing-dies.ashx#axzz2qvHzbO22) at least five people and injuring over 70. | January 2, 2014- January 8, 2014 | January 2, 2013- January 8, 2013 | **9.1 (16)** |  |  |
| **January 21, 2014** | An explosion ripped through Beirut's southern suburb of Dahiyeh shortly after 11:00 a.m. The explosion reportedly took place on the Martyr Ahmed Qassir road, some 30 meters away from where the January 2 blast took place. | January 21, 2014- January 27, 2014 | January 21, 2013- January 27, 2013  January 14, 2014- January 20, 2014 | **9.1 (16)** |  |  |
| **February 3, 2014** | A suicide bomber detonates an explosive belt inside a public minibus in Choueifat, south of Beirut, killing himself and another person and wounded 3 people, according to government sources. | February 3, 2014- February 9, 2014 | February 3, 2013- February 9, 2013  January 28, 2014 - February 2, 2014** | 14.3 (32) |  |  |
| **June 24, 2014** | A suicide bomber blows up a car near a security forces checkpoint, injuring 15 people and damaging a nearby cafe. | June 24, 2014- June 30, 2014 | June 24, 2013- June 30, 2013  June 17, 2014- June 23, 2014 | 6.2 (14) |  |  |
| *Only 6 days could be included in each of the case and control week, since January 2nd 2014 witnessed a violent event and was part of a case week, and hence January 2nd 2013 was part of the control week.  **Only 6 days could be included in the control week, since February 3rd witnessed a violent event and was part of a case week | | | | |  |  |
